# Genome-wide analysis of cell-Free DNA methylation profiling with MeDIP-seq identified potential biomarkers for colorectal cancer

**DOI:** 10.1186/s12957-022-02487-4

**Published:** 2022-01-22

**Authors:** Xin Zhang, Tao Li, Qiang Niu, Chang-jiang Qin, Ming Zhang, Guang-ming Wu, Hua-zhong Li, Yan Li, Chen Wang, Wen-fei Du, Chen-yang Wang, Qiang Zhao, Xiao-dong Zhao, Xiao-liang Wang, Jian-bin Zhu

**Affiliations:** 1 Department of General Surgery, Qingpu Branch of Zhongshan Hospital Affiliated to Fudan University, No. 1158 Gongyuan East Road, Qingpu District, Shanghai, 201700 China; 2 Department of General Surgery, Ruijin Hospital, Shanghai Jiao Tong University, School of Medicine, Shanghai, 200025 China; 3 Department of General Surgery, Shidong Hospital Affiliated to University of Shanghai for Science and Technology, Shanghai, 200433 China; 4 Department of Gastrointestinal Surgery, Huaihe Hospital of Henan University, Kaifeng, 475000 Henan China; 5 General Surgery, The People’s Hospital of Wuhai, Wuhai, 010600 Inner Mongolia China; 6 Digestive Internal, The People’s Hospital of Wuhai, No. 29 Huanghe East Street, Haibowan District, Wuhai, 010600 Inner Mongolia China; 7 Shanghai Center for Systems Biomedicine, Shanghai Jiao Tong University, Shanghai, 200240 China; 8 Department of General Surgery, Shanghai Pudong Hospital, Fudan University Pudong Medical Center, 2800 Gongwei Road, Pudong, Shanghai 201399 China

**Keywords:** Biomarkers, cfDNA, Colorectal cancer, MeDIP-seq

## Abstract

**Background:**

Colorectal cancer is the most common malignancy and the third leading cause of cancer-related death worldwide. This study aimed to identify potential diagnostic biomarkers for colorectal cancer by genome-wide plasma cell-free DNA (cfDNA) methylation analysis.

**Methods:**

Peripheral blood from colorectal cancer patients and healthy controls was collected for cfDNA extraction. Genome-wide cfDNA methylation profiling, especially differential methylation profiling between colorectal cancer patients and healthy controls, was performed by methylated DNA immunoprecipitation coupled with high-throughput sequencing (MeDIP-seq). Logistic regression models were established, and the accuracy of this diagnostic model for colorectal cancer was verified using tissue-sourced data from The Cancer Genome Atlas (TCGA) and Gene Expression Omnibus (GEO) due to the lack of cfDNA methylation data in public datasets.

**Results:**

Compared with the control group, 939 differentially methylated regions (DMRs) located in promoter regions were found in colorectal cancer patients; 16 of these DMRs were hypermethylated, and the remaining 923 were hypomethylated. In addition, these hypermethylated genes, mainly PRDM14, RALYL, ELMOD1, and TMEM132E, were validated and confirmed in colorectal cancer by using publicly available DNA methylation data.

**Conclusions:**

MeDIP-seq can be used as an optimal approach for analyzing cfDNA methylomes, and 12 probes of four differentially methylated genes identified by MeDIP-seq (PRDM14, RALYL, ELMOD1, and TMEM132E) could serve as potential biomarkers for clinical application in patients with colorectal cancer.

**Supplementary Information:**

The online version contains supplementary material available at 10.1186/s12957-022-02487-4.

## Background

Colorectal cancer is the most common malignancy and the third leading cause of cancer-related death worldwide [1–3]. Early diagnosis and treatment for colorectal cancer are crucial and often confer a good prognosis [4]. Colonoscopy is currently a common method of detecting colorectal cancer [5, 6]. However, colonoscopy is invasive and may cause serious complications [7]. It is generally believed that carcinoembryonic antigen (CEA) is the most characteristic serological marker for colorectal cancer [8], but the sensitivity of serum CEA is often low [9]. The fecal occult blood test (FOBT) is the most widely used method for colorectal cancer screening, but its sensitivity for the early detection of colorectal cancer is also low [8]. In reality, there are still many obstacles to the early diagnosis of colorectal cancer. If a novel biomarker can be developed for the early detection of colorectal cancer, it will have profound benefits for the general public.

Genetic and epigenetic aberrations of tumor cells occur at the initiation of tumorigenesis [10, 11]. DNA methylation is an important component of epigenetic modification [12]. Epigenetics has been a promising field in cancer research and includes the study of DNA methylation, which occurs in gene promoters [13]. Alterations in DNA methylation can affect gene expression in different ways; for example, the hypermethylation of tumor suppressor genes, especially in the gene promoter region, can lead to downregulation of the tumor suppressor gene and carcinogenesis, which play a key role in many cancers [2, 3]. Therefore, aberrantly methylated CpG sites located in the promoter region are considered promising cancer biomarkers.

When apoptotic or necrotic tumor cell lysis occurs, DNA fragments such as cfDNA are released into the bloodstream [14]. The detection of cfDNA could be helpful for early diagnosis and follow-up monitoring of tumors, as it has the advantages of being non-invasive and providing results in real time [15–17]. Many reports have pointed out that liquid biopsy studies, including cfDNA tests, and their clinical application may be helpful for tumor diagnosis, drug screening, efficacy evaluations, prognosis predictions, and tumor surveillance [14, 18–20]. Another type of DNA fragment released into the blood after apoptotic or necrotic tumor cell lysis is commonly referred to as circulating tumor DNA (ctDNA) [14, 21]. ctDNA has methylation patterns similar to those found in tumor cells [22].

The main experimental approaches for profiling genome-wide DNA methylation include whole-genome bisulfite sequencing (WGBS), reduced-representation bisulfite sequencing (RRBS), and MeDIP (methylated DNA immunoprecipitation coupled with high-throughput sequencing) [23]. Both RRBS and WGBS show substantial DNA degradation after bisulfite treatment, and WGBS is less cost-effective [23]. Recently, some scholars have reported that compared with other detection approaches, cfDNA methylated immunoprecipitation and subsequent high-throughput sequencing (cfMeDIP-seq) are more sensitive, accurate, and economical for the early diagnosis of tumors [24]. In recent years, there have been a few reports on the genome-wide detection of cfDNA methylation profiling by MeDIP-seq to screen potential tumor biomarkers. Xu et al. [25] identified hypermethylated DMRs in the promoter region that could be used as early diagnostic markers for lung cancer. Li et al. [26] identified hypermethylated DMRs located in promoter regions that completely overlapped with CpG islands and could be used for the non-invasive diagnosis of pancreatic cancer. To the best of our knowledge, there have been few reports on cfDNA methylation profiling by MeDIP-seq among colorectal cancer patients in China.

Therefore, in this study, we performed cfDNA methylation profiling in colorectal cancer patients by MeDIP-seq, followed by data analysis and validation.

## Methods

### Sample collection and cfDNA extraction

All colorectal cancer blood samples (*n* = 4) were obtained from patients with adenocarcinoma in Shanghai General Hospital, and control blood samples (*n* = 3) were obtained from healthy volunteers. Informed consent was obtained from all individuals. Specimens were collected and analyzed with the approval of the Ethics Committees of Shanghai General Hospital and Qingpu Branch of Zhongshan Hospital affiliated with Fudan University.

Blood from colorectal cancer patients and controls (~5 ml) was collected in tubes containing EDTA as the anticoagulant. Blood samples were centrifuged for 10 min at 1900×*g* and 4 °C. The plasma supernatant was carefully collected and centrifuged for 10 min at 16,000×*g* in a fixed-angle rotor at 4 °C. The plasma supernatant was carefully collected and frozen at − 80 °C.

Plasma cfDNA was extracted using the QIAamp Circulating Nucleic Acid Kit (Qiagen, 55114) according to the instructions. Qubit (Invitrogen) was used to analyze the concentration of cfDNA in plasma. An Agilent Bioanalyzer 2100 system was used to estimate the distribution of cfDNA size.

### MeDIP-seq library construction and sequencing

cfDNA was used for the preparation of the MeDIP-seq library with some modifications [27]. Briefly, we used the Illumina NEBNext Ultra II DNA Library Preparation Kit (NEB, E7645) and ligated ~ 50 ng of cfDNA to the Illumina adapter according to the manufacturer's instructions. The resulting library was denatured at 95 °C for 10 min, immediately incubated on ice for 10 min, and then immunoprecipitated with 5-methylcytosine (5-mC) monoclonal antibody (Epigentek, A-1014). The MeDIP DNA was amplified with Q5 high-fidelity DNA polymerase (NEB, M0491), and the amplified products were purified with AMPure XP beads (Beckman). The amplified libraries were evaluated using a Bioanalyzer 2100 system (Agilent Technologies), and deep sequencing was performed using an Illumina HiSeq 2000 system.

### Data processing and analysis

All qualified reads in the colorectal cancer patients’ and healthy individuals’ cfDNA MeDIP-seq raw data were mapped to the reference genome (Human hg38) using Bowtie (version 1.0.1) [28]. The MEDIPS analysis package (version 1.24.0) was used for the analysis and comparison of DNA methylation datasets between the patients and controls [29].

The 450K methylation array data (Illumina, San Diego, CA, USA) from normal colorectal tissue and colorectal cancer patient samples were obtained from the TCGA-COAD (colon adenocarcinoma) Samples Report (https://gdac.broadinstitute.org/runs/stddata__latest/samples_report/COAD.html) and GEO database (GSE42752, GSE52270, GSE77718). Independent-sample *t* tests were performed between normal samples and patient samples using the R statistical programming language (3.4.3, http://www.R-project.org) using the data processed with beta (β) values (proportion of the methylated signal over the total signal), and the hypermethylated target genes with a *p* value < 0.05 were selected.

## Results

### Whole-genome MeDIP-seq analysis of cfDNA

Plasma was collected from colorectal cancer patients (*n* = 4) and healthy controls (*n* = 3) for analysis in this study. The clinicopathological information of the patients is shown in Table 1. cfDNA was extracted from plasma using the QIAamp Circulating Nucleic Acid Kit.

**Table 1:** Clinicopathological information of colorectal cancer patients

Sample name	Gender	Age	Stage	Histology
J730	M	77	TMN III	Adenocarcinoma
J056	F	80	TMN III	Adenocarcinoma
J228	M	60	TMN IV	Adenocarcinoma
J474	M	68	TMN IV	Adenocarcinoma

*Note*: *J730*, *J056*, *J228*, and *J474* represent patients with colorectal cancer

cfDNA derived from colorectal cancer patients (*n* = 4) and healthy controls (*n* = 3) was used for the construction of the MeDIP-seq libraries, followed by next-generation sequencing.

An Illumina HiSeq 2000 system was used to sequence the MeDIP-seq libraries. On average, 27 million and 52 million raw sequencing reads were obtained from the colorectal cancer patient group and the control group, respectively. The proportions of reads matched with the reference genome (Human hg38) were 66.2% and 52.9%, respectively. After filtering out the repetitive reads, the patient group had an average of 15 million unique reads, and the control group had an average of 5 million unique reads (Table 2).

**Table 2:** Summary statistics of MeDIP-seq data

Sample	Number of total reads	Number of mapped reads	Total mapped read rate	Number of unique reads	Unique reads rate
J730	42,362,427	32,119,271	75.8%	26,454,305	82.4%
J056	29,652,736	21,442,064	72.3%	17,378,878	81.1%
J228	18,250,231	11,425,158	62.6%	9,003,634	78.8%
J474	19,015,432	10,281,561	54.1%	8,097,962	78.8%
C1	46,505,740	16,686,432	35.9%	2,089,072	12.5%
C2	18,918,360	11,095,194	58.7%	5,085,484	45.8%
C3	91,305,808	58,482,718	64.1%	8,453,424	14.5%

*Note*: *J730*, *J056*, *J228*, and *J474* represent patients with colorectal cancer; *C1*, *C2*, and *C3* represent healthy controls

### Distinctive cfDNA methylation patterns between colorectal cancer patients and healthy controls

**Fig. 1:**
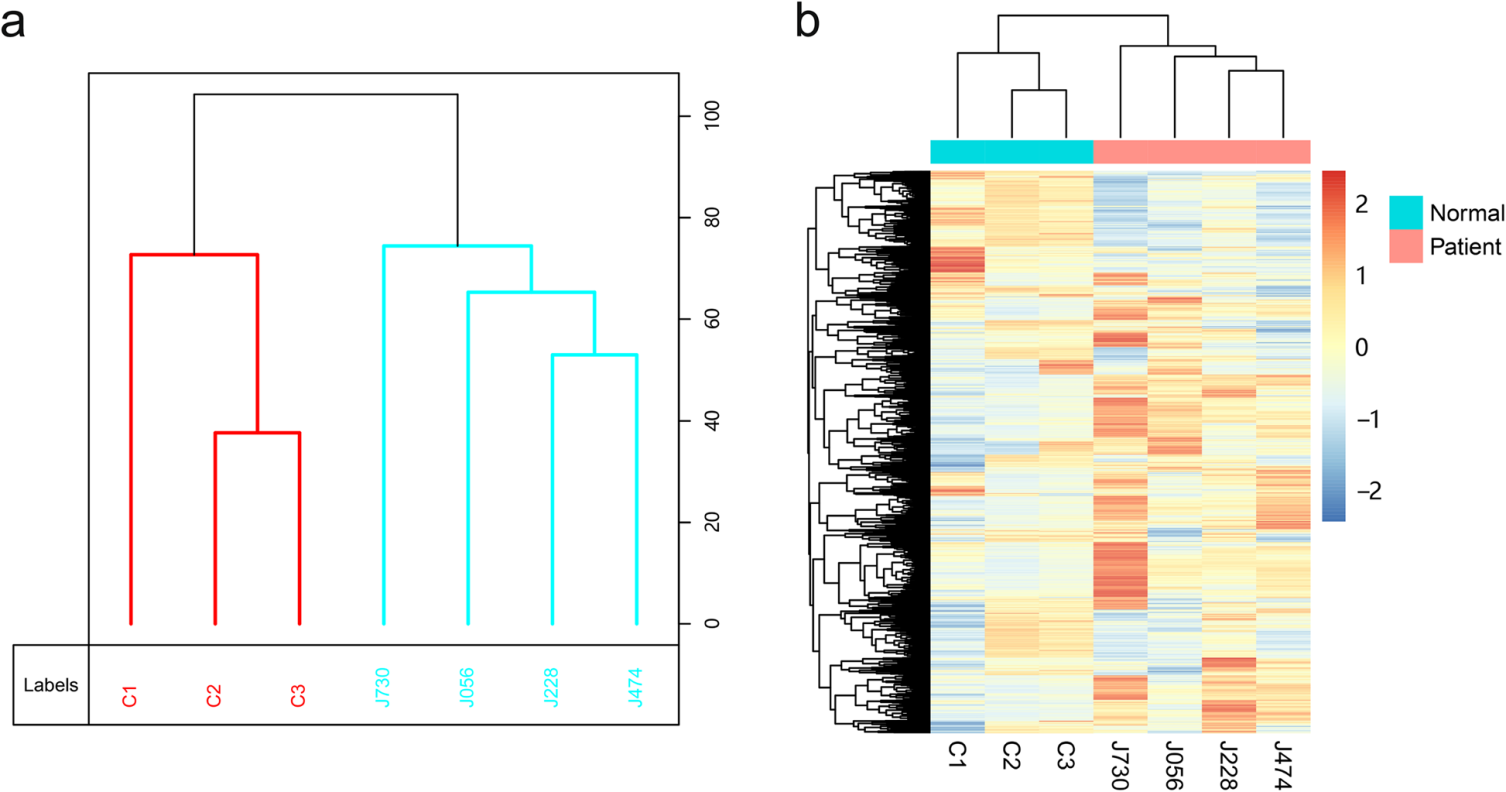
The cfDNA methylation patterns derived from MeDIP-seq datasets between colorectal cancer patients and controls. **a** Heuristic cluster analysis of methylation profiling between patients and controls. **b** Unsupervised cluster analysis of the genome-wide methylation profiling in patients and controls

To determine the overall cfDNA methylation patterns in the patients and healthy controls, we performed heuristic cluster analysis and unsupervised cluster analysis on cfDNA MeDIP data from colorectal cancer samples and normal samples, respectively. Through heuristic cluster analysis, we found that the methylation patterns were distinctive between the patient group and the control group (Fig. 1a). Genome-wide unsupervised cluster analysis also confirmed distinct methylation patterns between the two groups (Fig. 1b).

### Differentially methylated regions (DMRs) in colorectal cancer patients

**Fig. 2:**
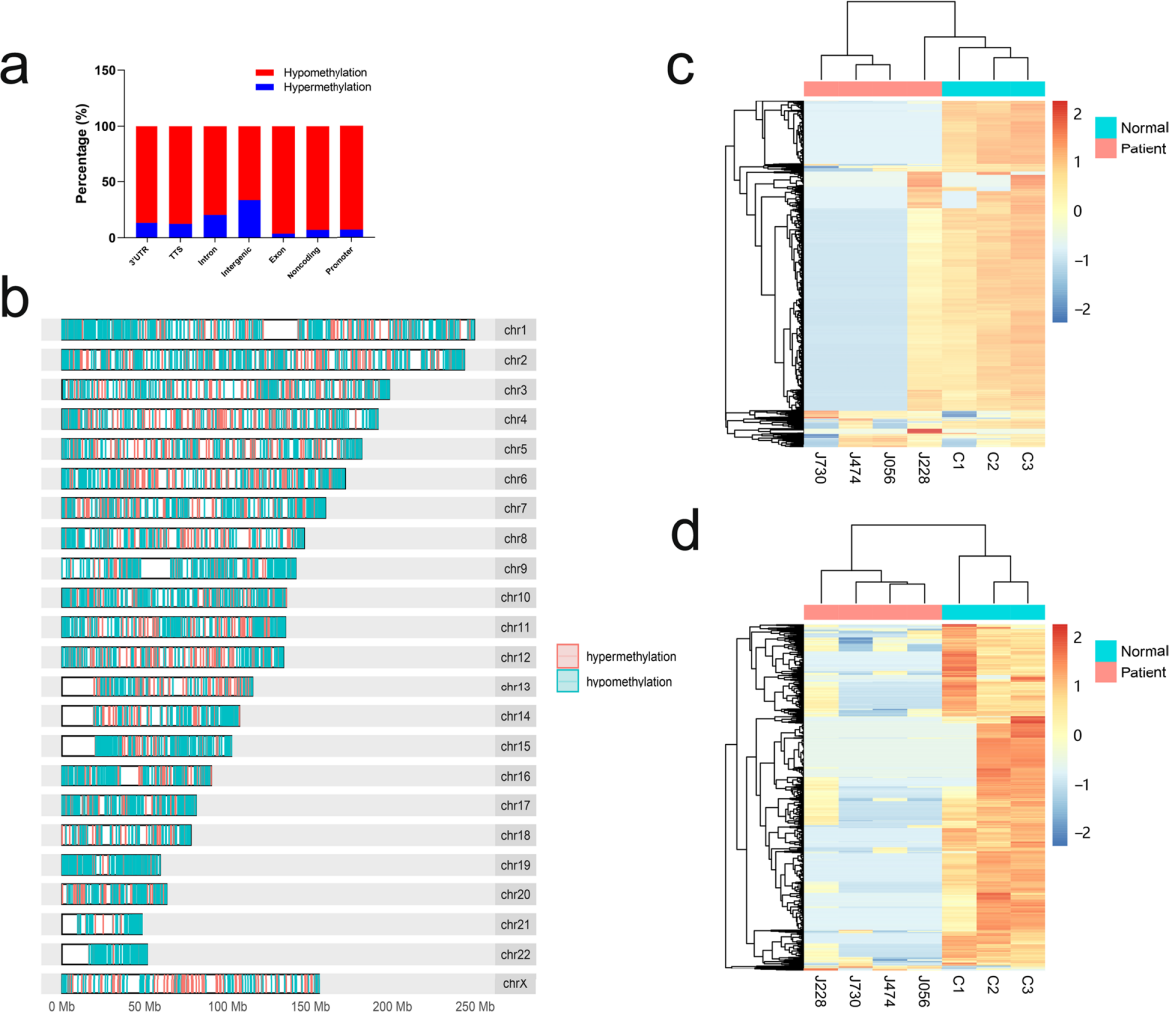
Differentially methylated regions in patients and controls. **a** The genomic distributions of hypomethylated and hypermethylated DMRs in introns, intergenomic, exons, non-coding, promoters and other regions. **b** The distribution of DMRs mapped to the whole genome on different chromosomes in patients. **c** Heat map of total 8398 DMRs, including 1875 hypermethylated and 6523 hypomethylated. **d** Heat map of DMRs located in promoter regions in patients and controls, including 16 hypermethylated and 923 hypomethylated

With the help of the MeDIPS analysis package, a total of 8398 DMRs were obtained from the genome-wide distribution of patients (*p* value < 0.05). Among these DMRs, 1875 (22.3%) were hypermethylated, and 6523 (77.7%) were hypomethylated (Supplementary Table 1). We examined the genomic distributions of the hypomethylated and hypermethylated DMRs and found that the proportion of hypermethylated DMRs was higher in the intergenic and intronic regions (Fig. 2a). The distribution of DMRs mapped to the whole genome on different chromosomes is shown in Fig. 2b. The 8398 DMRs exhibiting distinct patterns between colorectal cancer patients and normal controls are shown in Fig. 2c.

Hypermethylation in the promoter region of tumor suppressor genes is known to be positively correlated with the occurrence and development of tumors [21, 30]. Therefore, we further analyzed DMRs and identified 939 DMRs located in promoter regions (Fig. 2d and Supplementary Table 2), including 16 hypermethylated regions and 923 hypomethylated regions. Furthermore, these 939 DMRs in the promoter regions also exhibited distinct patterns between the patients and the controls.

### Validation of differentially methylated genes by using publicly available DNA methylation data

As mentioned above, we found that 16 of the DMRs located in the promoter region were hypermethylated, so we next wanted to determine whether the methylation levels of these corresponding genes could help to distinguish colorectal cancer patients from healthy individuals.

After annotating 16 DMRs with hypermethylated promoter regions, 13 genes were obtained, and their corresponding promoter region microarray probes were screened. During the screening process, probes located in the sex chromosome and the 3′UTR regions and the gene body regions were excluded, as were the SNP-related probes. Only the probes located in the UCSC (University of California Santa Cruz)_CpG_Island regions were retained, so a total of 12 probes were used (Supplementary Table 3). The corresponding genes of the 12 probes mentioned above are PRDM14, RALYL, ELMOD1, and TMEM132E.

**Fig. 3:**
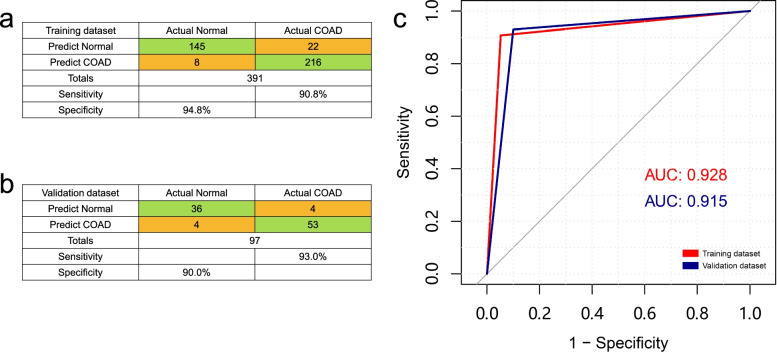
Diagnostic predictive models and receiver operating characteristic (ROC) curves for colorectal cancer. **a**, **b** Confusion matrix built from the diagnostic predictive models in training (**a**) and validation (**b**) dataset. COAD, colon adenocarcinoma. **c** ROC curves and the associated area under the curve (AUCs) of the training and validation dataset

The 450K methylation array data were obtained from TCGA and GEO datasets, including both colorectal cancer patient samples (*n* = 295) and normal colorectal tissue samples (*n* = 193). Based on the aforementioned 12 probes, the predictive model of the logistic regression algorithm was established, and the 488 original data points were divided into the training dataset and validation dataset at a ratio of 4:1. The predictive ability of the model in the two datasets is shown in Fig. 3. According to the receiver operating characteristic (ROC) curves shown in the figure, the areas under the curve (AUCs) of the training dataset and the validation dataset were 0.928 and 0.915, respectively. Figure 3a, b shows the confusion matrix of the training dataset and the validation dataset, respectively. This suggested high validity for the diagnosis of colorectal cancer based on methylation levels of the 12 probes described above.

**Fig. 4:**
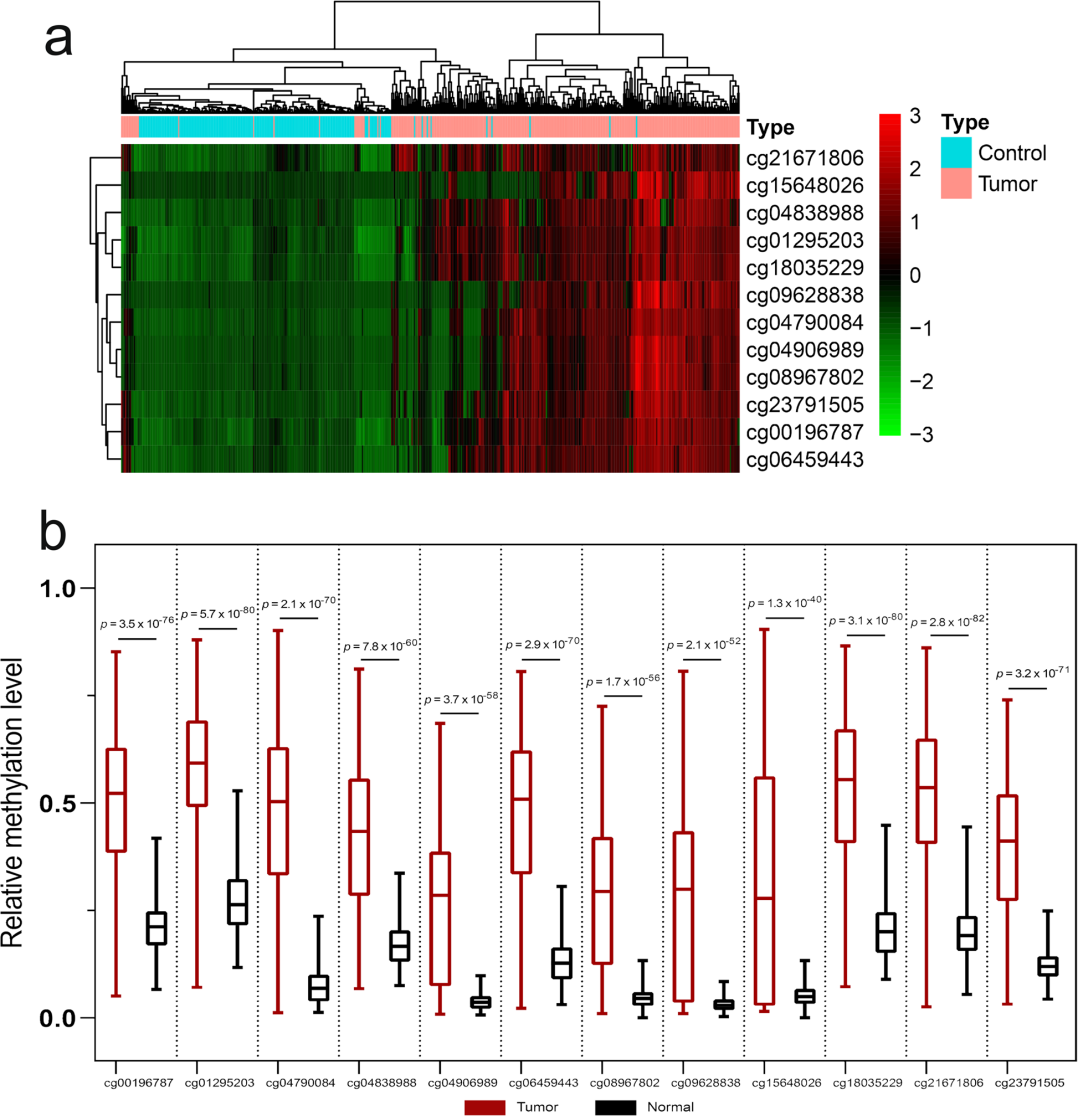
Validation of hypermethylated genes by using publicly available DNA methylation data. **a** Unsupervised cluster analysis of these 12 probes extracted from the 488 cases of 450K methylation array dataset. **b** The comparison of methylation level between tumor and normal tissue of the 12 selected probes. All *p* values < 0.05

We then extracted the 12 probes for unsupervised cluster analysis based on the 488 data points in the 450K methylation array dataset, and the results showed that the methylation data of the aforementioned 12 probes were distinct between tumor and normal tissues in general (Fig. 4a). We also compared the methylation levels of the aforementioned 12 probes between normal colorectal tissue and colorectal cancer patient tissue samples in the dataset, and we found that the methylation levels of the aforementioned 12 probes were significantly different (*p* value < 0.05). Compared with normal colorectal tissue, the methylation level of the 12 probes in the tumor tissue was hypermethylated (Fig. 4b). These results suggest that detecting the methylation levels of these 12 probes and their corresponding genes is helpful for the diagnosis of colorectal cancer.

## Discussion

Abnormal patterns of DNA methylation, including the hypermethylation of gene promoter regions accompanied by gene silencing, play a key role in many types of cancer [13]. When apoptotic or necrotic tumor cells lyse, they release DNA fragments comprising cfDNA into the bloodstream [14]. Moreover, the methylation pattern of cfDNA in peripheral blood is similar to that found in tumor cells [22]. In this study, we performed a genome-wide epigenetic profiling assessment of patients with colorectal cancer using MeDIP-seq technology to screen for potential cfDNA biomarkers. Our analysis revealed global changes in cfDNA methylation patterns in colorectal cancer patients. We found 8398 DMRs in cfDNA collected from patients with colorectal cancer at the genome-wide level, among which 1875 (22.3%) were hypermethylated and 6523 (77.7%) were hypomethylated. When we focused on DMRs located in the promoter region, 16 (1.7%) were hypermethylated, and 923 (98.3%) were hypomethylated. This finding suggests that demethylation is widespread in cancer patients at the genome-wide level [31], with a higher proportion of hypomethylation observed in promoter regions. Studies have shown that DNA demethylation plays an important role in activating specific gene expression and the initiation of reprogramming [32].

After screening and annotating 16 hypermethylated DMRs in the promoter region, we obtained 12 probes from 4 differentially methylated genes, including PRDM14, RALYL, ELMOD1, and TMEM132E. Many reports have described the function of these genes: PRDM14 has been reported to be hypermethylated in lung cancer and has high accuracy in the diagnosis of lung cancer [33, 34]. Studies have also shown that PRDM14 has several hypermethylated CpG sites in African-American colorectal cancer patients by using RRBS [35]. Meanwhile, we used MeDIP-seq technology to study cfDNA in the peripheral blood of Chinese patients with colorectal cancer. Although there were differences in the research methods, species, and specimens used, we obtained consistent results. RALYL has been reported to be downregulated in clear cell renal cell carcinoma, and its reduced expression is associated with poor prognosis [36], which means that it could serve as a tumor suppressor gene. Li et al [37] identified TMEM132E mutation as the most likely cause of autosomal recessive non-syndromic hearing loss by whole-exome sequencing. Johnson et al. [38] found that mutations in ELMOD1 may cause cochlear hair cell dysfunction, eventually leading to deafness in mice. Studies on the methylation of the last three genes in colorectal cancer have been rarely reported and are worthy of further study and verification.

Subsequently, to evaluate the diagnostic value of hypermethylated genes in colorectal cancer, methylation data were obtained from publicly available DNA methylation datasets due to the lack of cfDNA methylation data in public datasets. A predictive model of the foresaid 12 probes was constructed to confirm its high validity. Based on the diagnostic predictive model, we have demonstrated in the results section that we can effectively distinguish colorectal cancer patients from healthy controls by comparing their methylation levels in peripheral blood cfDNA. According to the training cohort (AUC = 0.928) and validation cohort (AUC = 0.915), the diagnostic prediction model could still distinguish colorectal cancer tissues from normal tissues. These results provide new methylation biomarkers for the early diagnosis of colorectal cancer. These findings indicate that the methylated genes that were identified from cfDNA derived from colorectal cancer patient plasma may have clinical application value. Therefore, cfDNA combined with MeDIP-seq, as a non-invasive and real-time diagnostic technique, is expected to be an effective method for the early clinical diagnosis of a variety of cancers [25, 26].

## Conclusions

In summary, the results of our study indicate that MeDIP-seq can be used as an optimal approach for analyzing cfDNA methylomes, and 12 probes of four differentially methylated genes identified by MeDIP-seq (PRDM14, RALYL, ELMOD1, and TMEM132E) could serve as potential biomarkers for clinical application in patients with colorectal cancer.

## Supplementary Information

**Additional file 1: Supplementary Table 1.** Genome DMRs identified in cfDNA of colorectal cancer patient plasma.

**Additional file 2: Supplementary Table 2.** Promoter DMRs identified in cfDNA of colorectal cancer patient plasma.

**Additional file 3: Supplementary Table 3.** 12 Illumina HM450K BeadChip Array probes corresponding to 16 candidate DMRs.

## Data Availability

The datasets used during the present study are available from the corresponding author upon reasonable request.
